# The zone of latent solutions and its relevance to understanding ape cultures

**DOI:** 10.1007/s10539-020-09769-9

**Published:** 2020-10-11

**Authors:** Claudio Tennie, Elisa Bandini, Carel P. van Schaik, Lydia M. Hopper

**Affiliations:** 1 Department for Early Prehistory and Quaternary Ecology, University of Tübingen, Tübingen, Germany; 2 Department of Anthropology and Anthropological Museum, University of Zürich, Zürich, Switzerland; 3 Lester E. Fisher Center for the Study and Conservation of Apes, Lincoln Park Zoo, Chicago, IL USA

**Keywords:** Zone of latent solutions, Ape culture, Chimpanzee, Individual learning, Copying social learning, Imitation, Socially mediated reinnovations, Copying-dependent forms

## Abstract

The zone of latent solutions (ZLS) hypothesis provides an alternative approach to explaining cultural patterns in primates and many other animals. According to the ZLS hypothesis, non-human great ape (henceforth: ape) cultures consist largely or solely of latent solutions. The current competing (and predominant) hypothesis for ape culture argues instead that at least some of their behavioural or artefact forms are copied through specific social learning mechanisms (“copying social learning hypothesis”) and that their forms may depend on copying (copying-dependent forms). In contrast, the ape ZLS hypothesis does not require these forms to be copied. Instead, it suggests that several (non-form-copying) social learning mechanisms help determine the frequency (but typically not the form) of these behaviours and artefacts within connected individuals. The ZLS hypothesis thus suggests that increases and stabilisations of a particular behaviour’s or artefact’s frequency can derive from socially-mediated (cued) form reinnovations. Therefore, and while genes and ecology play important roles as well, according to the ape ZLS hypothesis, apes typically acquire the forms of their behaviours and artefacts individually, but are usually socially induced to do so (provided sufficient opportunity, necessity, motivation and timing). The ZLS approach is often criticized—perhaps also because it challenges the current null hypothesis, which instead assumes a requirement of form-copying social learning mechanisms to explain many ape behavioural (and/or artefact) forms. However, as the ZLS hypothesis is a new approach, with less accumulated literature compared to the current null hypothesis, some confusion is to be expected. Here, we clarify the ZLS approach—also in relation to other competing hypotheses—and address misconceptions and objections. We believe that these clarifications will provide researchers with a coherent theoretical approach and an experimental methodology to examine the necessity of form-copying variants of social learning in apes, humans and other species.

## Introduction

Despite the continuously growing body of research on the behavioural repertoires of non-human great apes (henceforth: apes), the necessary mechanisms behind the acquisition of their various behavioural and artefact forms^1^ are still a matter of debate. Many have argued that apes acquire their behavioural and artefact forms through similar social learning mechanisms to those often relied upon by humans: i.e. that apes, like humans, depend on *form*-*copying* social learning mechanisms (henceforth: copying; e.g., Whiten et al. 1999, 2001; Gruber et al. 2015). Here, we call this the “copying social learning hypothesis.”

One ape behavioural form that is often argued to be copied, and reliant on form-copying variants of social learning, is nut-cracking (with tools) by wild chimpanzees. Although nuts, and the tools required for nut-cracking (i.e. wooden or stone hammers and anvils), can be found across chimpanzee home ranges in Africa, only some communities use tools to crack nuts. Predominantly, only chimpanzees in West Africa perform nut-cracking, with the N’Zo-Sassandra river separating the nut-cracking populations from those who do not (Boesch et al. 1994). The variance in whether, and how (i.e. the form, as the expression of know-how; Tennie et al. 2017), chimpanzees crack nuts with tools has been said to be a product of form-copying, in particular of imitation (Whiten et al. 1999). However, in 2006 researchers reported nut-cracking in chimpanzees in Cameroon, 1700 km away from the West African populations (although note that this was an indirect observation; Morgan and Abwe 2006). Furthermore, several captive primate populations (that had likely not observed the nut-cracking behavioural form) simply “reinnovated” nut-cracking in experimental studies (Visalberghi 1987; Marshall-Pescini and Whiten 2008; Bandini et al., in review; but see also Boesch 1996). Taken together, these instances of *copying*-*independent (re*-*)innovations* of the underlying form suggest that social learning, of *any* type (including copying, such as imitation), may not be necessary for the acquisition of the form of nut-cracking by individual chimpanzees, although the geographic pattern (as with the *Neesia* tool use in wild orangutans: van Schaik 2009) indicates that its’ innovation is not easy and may take a long time.

The ape copying social learning hypothesis continues to be pervasive in the literature as a null hypothesis, despite growing evidence that many species of apes (and monkeys) can acquire behavioural forms without requiring any copying variants of social learning (Lefebvre 1986; Custance et al. 1999; Bernstein-Kurtycz et al. 2020; Dindo et al. 2008; Tennie et al. 2008; van de Waal et al. 2010; Allritz et al. 2013; Menzel et al. 2013; Reindl et al. 2016; Kis et al. 2015; Bandini and Tennie 2017, 2019; Neadle et al. 2017; ), and despite the lack of evidence for copying variants of social learning—especially regarding behavioural forms—in unenculturated/untrained apes (Tomasello et al. 1987; Tennie et al. 2012, 2020; Clay and Tennie 2017; Henrich and Tennie 2017—see also Bohn et al. 2020).^2^ Thus, an alternative account is needed to explain ape culture.

In 2009, Tennie et al., proposed such an alternative account: the zone of latent solutions (ZLS) hypothesis. Whilst this approach can be used to potentially explain culture in any animal species (though it may not fit every culture),^3^ the more specific “ape ZLS hypothesis” states that ape culture arises—and is maintained—by social learning variants that do not produce copies of behavioural forms (i.e., non-form-copying social learning, henceforth also: “non-copying”). These non-copying social learning mechanisms still matter—at the right time and in the right circumstances—as they can facilitate individual reinnovation and, by doing so, regulate (and potentially encourage) the frequencies of behavioural forms in populations but without leading to (or requiring) copies of behavioural form. Within this account, *socially*-*mediated individual learning* of forms drives the likelihood of individual acquisition of behavioural forms (Bandini and Tennie 2017). If the ape ZLS hypothesis is true, ape behavioural repertoires would be the sum of purely individually produced forms plus socially-mediated reinnovated forms (SMR; Bandini and Tennie 2017). This would be in stark contrast to the majority of modern human cultural behavioural (and artefact) forms, which, arguably, depend on copying (culture-dependent traits, Reindl et al. 2017; or, more specifically, what we refer to here as: copying-dependent forms).^4^ Copied form is of high importance to human culture (e.g. Richerson and Boyd 2005; Tomasello 1999; Tennie et al. 2009; but see also section “ZLS as related to cultural epidemiology” and Sperber 2000; Morin 2016). Examples of human behavioural forms that most likely depend on copying include many culturally evolved rituals, dances, languages and technological knowledge that cannot be reinnovated by individuals without cultural access to these or underlying forms (see more discussion on this below). That is, these forms will not be produced by those on “cultural islands” that lack access to these forms (Boyd and Richerson 1996; Tomasello 1999).^5^ The copying social learning hypothesis therefore fits much of human culture, but—according especially to the ape ZLS hypothesis—not many, if any, aspects of ape culture.^6^

The ape ZLS hypothesis proposes that each ape can potentially acquire “target” (e.g. cultural) behavioural forms from its ZLS individually (e.g., Whiten et al. 1999; van Schaik et al. 2003). These behavioural forms are assumed to be ‘latent solutions,’ and within the species’ potential behavioural-form repertoire (its ZLS). These behavioural forms can emerge when the individual experiences the appropriate ecological and social circumstances, and is in the right developmental and motivational state (Bandini and Tennie 2018; cf. Lehner et al. 2010). Although the behavioural forms within a species’ ZLS emerge via individual learning, non-copying variants of social learning often facilitate their acquisition (by, for example, attracting the individual to the area (“know-where” Bandini et al. 2020) in which others are performing the behaviour;^7^ see Whiten et al. 2004 for an overview of the various social learning mechanisms). Thus, non-copying social learning mechanisms help not only create, but also sustain, the resultant population-level behavioural repertoires typically described as ape cultures (e.g., chimpanzees: Whiten et al. 2001; orangutans: van Schaik et al. 2003; gorillas: Robbins et al. 2016). Thus, social learning is dispensable for the acquisition of behavioural forms, but it can greatly increase the individual likelihood of even specific form reinnovation^8^ and, with it, help produce and maintain the observed frequencies of particular latent solutions in populations (Tennie et al. 2009, 2020; Bandini and Tennie 2017). So, while apes appear to observe the behavioural forms of other group members (Yamanashi et al. 2016; Schuppli et al. 2016; Whiten and van de Waal 2017), these situations may merely activate non-copying social learning mechanisms that simply stimulate (via various cues) those affected to reinnovate similar behavioural forms from within their ZLS, rather than relying on, e.g., action form copying (though this situation often creates an illusion of copying to observers). Therefore, the ZLS hypothesis is an account of how even population differences (often referred to as culture proper) can come about without the need for form copying variants of social learning.^9^ Additionally, given the lack of evidence for spontaneous action form copying in ecologically-representative, i.e. untrained and unenculturated, apes (Henrich and Tennie 2017), the ape ZLS hypothesis suggests that apes are restricted to their ZLS, at the very least when it comes to action-form-based cultures (e.g., gestures; action components of tool use; Motes-Rodrigo and Tennie in review). This view is reinforced by the finding that naïve, ecologically-representative apes individually reinnovate wild-type behavioural forms (see above, including tool-use behavioural forms; Bandini and Tennie 2017, 2019).

By re-describing ape culture, the ZLS approach also provides an explanation for the difference between human and ape cultures. Whilst modern humans also have a ZLS (i.e. behavioural and artefact forms they can independently reinnovate without requiring form copying; Neldner et al. 2020; Reindl et al. 2016), unlike apes, humans can go beyond their ZLS through form copying variants of social learning (including action copying: Tomasello 1999), towards achieving cumulative culture of forms—via the ratchet effect (Tomasello 1999; Tennie et al. 2009; Dean et al. 2012).^10^ This is because form-copying allows cumulative culture of these forms—a cultural evolution of know-how (Tennie et al. 2017)—which eventually produces copying-dependent forms,^11^ (e.g., Shakespeare‘s works must be copied and cannot be simply reinnovated in their precise form). In contrast, the ZLS approach claims that the lack of form copying by apes—especially of action forms—prevents them from having action copying-dependent forms (compare Tennie et al. 2009, Neadle et al. 2017, 2020). The difference in social learning mechanisms available to a species (due to various types of evolution, including cultural evolution, compare Heyes 2018) is hypothesized to result in variation in the types of cultures that are produced. The ZLS view therefore contrasts and highlights what the presence or absence of form-copying means for the long-term evolution of cultures.

**Fig. 1:**
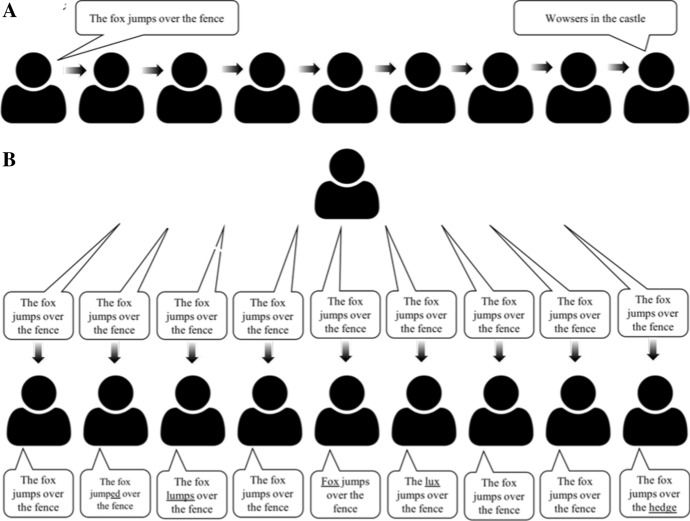
**a** The classic telephone game, in which the original sentence’s form is copied along players, often resulting in the last individual receiving a completely different sentence form than what was originally whispered by the first participant. **b** The ‘socially mediated reinnovation game’ in which the sentence form is repeated anew to each participant by an independent whisperer, without any form copying between individuals. This game results in each individual producing almost exactly the same sentence form—creating an illusion of copying to outside observers

The ZLS approach requires us to view ape culture from a new perspective and to question long held assumptions (such as that “apes ape”). To illuminate the differences between ape and modern human culture even further, we hope it will be helpful to envision the differences in a human context. Imagine the classic “telephone game” in which a group of people whisper a predefined message to each other along a chain, copying the form—here the evolving message—from one player to the next. When done correctly, this process quickly leads to a humorous cultural evolution of the initial message (the form) due to copying errors. If the predefined message is “The fox jumps over the fence”, after e.g., nine players the final message form may be “wowsers in the castle” or “chicken soup” or indeed pretty much anything at all (Fig. 1a). The telephone game speeds up cultural evolution through an intentional and artificial increase of copying error in a cultural series of form-copying. Let us now consider a new variant of this game that illustrates the way in which the ape ZLS hypothesis sees ape (and many other animal) culture. In this variant—the “socially mediated reinnovation game”—nothing except a specific cue passes from player to player (e.g., a tap on the next shoulder does the job, or a tap at different, but specific, places when there are more than one reinnovations that can be triggered). Here, the whispering is done instead by *independent* whisperers standing behind the players—who, once cued, each whisper the message’s form (e.g., “The fox jumps over the fence”) in the ear of their corresponding player (one whisperer per player) who do not speak out loud the message until the end of the game. Here, there is no copying *between* players (Fig. 1b)—the message is not copied and therefore its form cannot culturally evolve along players. Unsurprisingly, this second game leads to a boring outcome (as we have repeatedly shown informally by playing both games with students in lectures). Generally, the outcome is that the original message remains pretty much intact for each player—though there is also some variability around the message’s form (e.g., the original “The fox jumps over the fence” may turn into “The fox jump**ed** over the fence” in, say, player 3, or into “The fox jump**s** over the fence” in player 8 or into “The fox jumps over the **pence**” in player 20 etc.). This new outcome (Fig. 1b) differs from the familiar one (Fig. 1a) because only the original telephone game consists of repeated cultural transmission chains of (error-prone) copying, mimicking the logic in which modern human cultural forms can accrue incremental changes across generations.^12^ It is noteworthy that the only one of the two games that actually contains copying social learning between participants (the telephone game) leads to *less* similarity among the participants’ behaviour (the messages) over time/generations, than the game variant that does *not* contain copying social learning between the participants (the socially mediated reinnovation game). This illustrates that merely using “similarity judgements”^13^ and automatically equating them with processes of copying is dangerous in that it can lead to illusions of copying where little or even no form copying actually took place (compare also to Morin 2016). Yawning contagion is more like the individual learning game than the telephone game, for example (Yoon and Tennie 2010)—but to an observer it may look (at first) more like a telephone game. Copying can be a (powerful) illusion in observers of cued reinnovation situations.

Our proposed variation of the telephone game (Fig. 1b) is roughly the equivalent of latent solutions increasing in frequency in a wild ape population:^14^ here, instead of continuous and dependent copying, each participant independently and individually recreates the output signal form anew (corresponding to the individual whispers in the game)^15^ rather than copying the form from other players.^16^ If ape behavioural repertoires really consist of latent solutions, their acquisition process should more closely follow the second game (the socially mediated reinnovation game), in which small socially mediated information (e.g. a tap, or “you are next”, or, e.g. a stone next to a nut) merely facilitate/activate the likelihood of individual acquisition of the corresponding form of the cued behaviour. Overall, however, the general behavioural form (in our game represented by the verbal message) will remain *highly similar across individuals*, creating and maintaining the population-wide patterns of behavioural repertoires observed in wild apes, and explaining why naïve individuals are able to reinnovate the same behavioural forms as their wild counterparts without having access to target forms (and without action form copying abilities; e.g. Tennie et al. 2008; Allritz et al. 2013; Menzel et al. 2013; Bandini and Tennie 2017; Neadle et al. 2017). We therefore surmise the same principles are at play in wild populations—explaining differences in form expression frequencies across populations—though these often are (wrongly) interpreted as evidence of form copying.

Since its publication in 2009, the ZLS hypothesis, and especially the ape ZLS hypothesis, has been heavily criticized. To respond to these critiques, here we address and clarify the main misconceptions and objections to the ZLS hypothesis (see also Tennie et al. 2020). We will also explain the differences to alternative approaches and argue that the ape ZLS approach provides a valid alternative explanation for the behavioural frequencies observed in wild-representative apes.

## The role of social learning in the acquisition of behavioural forms, according to the ZLS

Some authors have misinterpreted the ape ZLS hypothesis to suggest that it discounts any role of social learning for wild apes (Gruber et al. 2012; Haidle and Schlaudt 2020). While there can be ape behavioural forms that do not depend on social learning (not even in their frequencies; e.g. breathing), the more typical case—and the case underlying wild ape cultures, is one in which social learning plays a role, sometimes even a large one. Indeed, in all descriptions of the ZLS approach (including in the first, i.e. Tennie et al. 2009) non-copying social learning mechanisms are an important and even necessary part in explaining ape cultures. Such learning mechanisms (e.g., stimulus and local enhancement, exposure etc.; Arbilly and Laland 2014; Heyes and Pearce 2015; Heyes 1994; Zuberbühler et al. 1996) can, and often do, greatly increase the likelihood of specific individual reinnovations across connected individuals, thus increasing (and eventually stabilising) the frequency of the affected behavioural form within a population (i.e. socially-mediated reinnovation; Bandini and Tennie 2017).^17^ Via exposure to others practicing the target behavioural form (including the information where and when and with what they do so—socially provided specific cues for reinnovations of the very same form in others), individuals are much more likely to reinnovate the same behavioural or artefact form themselves (compare also “cultural founder effects”; Tennie et al. 2009). For example, observer Z may be more likely to interact with object A after having seen demonstrator X interact with object A, or a functionally similar object (a stick, or even a wooden hammer instead of a stone hammer). Yet, the actual behavioural form observer Z develops would not necessarily have to be the same as the one adopted by demonstrator X—but when it is, this does not necessarily mean that the form was copied (see Fig. 1b). Therefore, the ape ZLS hypothesis explicitly claims that social learning plays a role (and even a major role) in ape culture. Additionally, in doing so, the ZLS approach does not negate the existence of ape cultures (it predicts them). Instead, the ZLS adheres to a minimal definition of culture, which in essence equates social learning (of any variant) with culture (Neadle et al. 2017). While some definitions of culture require additional factors, such as temporal stability in the expression of behavioural forms, even this addition does not pose a problem for the ZLS view of ape cultures—as ape cultures can sometimes last thousands of years (Mercader et al. 2002, 2007). Apes have culture, but the effect of social learning is not on the copying of the behavioural form but on the frequency in which these forms appear.

Further evidence for the view that apes’ expression of their behavioural forms is often socially mediated comes from the fact that (currently) there is no clear data showing that ape behavioural forms have changed throughout time (that they cumulating form modifications, as required for cumulative culture of forms; Mesoudi and Thornton 2018). An archaeological excavation of a chimpanzee nut-cracking site yielded nut-cracking materials dated to 4300 years of age, identical in behavioural form affordance to the tools used today by modern chimpanzees (Mercader et al. 2002, 2007). If the form of nut-cracking was being copied between chimpanzees, we would expect to see some changes, even random changes (drift), if just by (unavoidable) copying error alone (Eerkens and Lipo 2005).^18^ Yet, the consistency (or stasis) in the behavioural form (i.e., the mechanical actions and results of placing the nut on an anvil-like structure (when it isn’t there already) and then lifting a hammer and forcibly hitting it onto the nut) for at least 4300 years suggests that chimpanzees within and across generations are all individually reinnovating this core form of the behaviour. Yet, the frequency of these reinnovations is stabilized and mediated by non-copying forms of social learning (as in our Fig. 1b). Though note that temporal stability alone cannot fully exclude the presence of form-copying skills. Other factors can keep certain forms in check, as they may counteract copy error. For example, there is a type of bone tool still in similar form-use today as it was many thousands years ago by Neandertals (so-called lissoirs; Soressi et al. 2013), where it seems difficult, even today, to further improve their form and use. That is, there can be environmentally prescribed/channelled optima that prevent form modifications from cumulating, which may at times mask a potential presence of form-copying abilities. Theoretically, there could also be genetic fixations (e.g. Corbey et al. 2016). Yet, currently there is no reason to assume that such form-constraining factors are widespread, and so, everything else being equal and without independent data providing evidence for form-copying abilities, stasis is most compatible with an absence of form copying.^19^

In the first formulation of the ape ZLS hypothesis (presented by Tennie et al. 2009), the apparent lack of copying variants of social learning mechanisms (termed “high-fidelity social learning”^20^ and “process copying” in this original paper) in ecologically relevant apes was a core argument for the supposed difference between human and ape culture. In the original paper, the main copying mechanism explored was imitation. Responding to Tennie et al. (2009), Moore (2013, p. 894) argued: “to say that a behaviour is a latent solution is therefore not, on current formulation, to extend the culture debate beyond the claim that apes do not learn through imitation.” While it is true that the early description of the ZLS approach relied on the exclusion of imitation (although already focusing on its main defining form-copying component, i.e., action copying) as a learning mechanism in ZLS-bounded species, the lack of copying variants of teaching in apes (and other factors and predictions) was also already described (Tennie et al. 2009). Thus, the critique by Moore (2013) was not relevant to the original formulation of the ZLS. Additionally, there have been further extensions and clarifications of the ZLS approach since 2009—which we refer to above and below.

Tennie et al. (2009) claimed that only “process copying” (such as imitation, as in goal-sensitive action copying) can allow for the evolution and maintenance of trait forms that no individual could reinnovate on their own and therefore must be copied (i.e., copying-dependent forms). However, new empirical evidence demonstrates that variants of emulation learning (subsumed under a “product copying” label in Tennie et al. 2009 and see also Tomasello 1998, 1999) may also produce/transmit cumulative culture—at least in human adults (Caldwell and Millen 2008; Caldwell et al. 2012; Reindl et al. 2017). Yet this conclusion does not seem to apply across all possible cases—there seem to be even technological tasks for which action copying is still required (Wasielewski 2014). Note however, more generally, that emulation learning can indeed—at least potentially—transmit the form of artefacts (their outer form as well as their inner form and/or hierarchical relations). This is how furniture building instruction manuals often work—form instructions minus actions—and these can indeed help lead to correct assemblages of even complex artefact forms. Thus, other variants of social learning (including some variants of emulation) can, at least sometimes, be sufficient to lead to copying-dependent forms. Given that it is widely accepted that apes can emulate, it is possible that apes could have developed copying-dependent forms via emulation learning. However, concrete evidence for copying-dependent forms in ape culture is still lacking and past emulation tasks did not often test for copying beyond the species’ ZLS. However, in one study, when experimentally tested on the ability to copy a copying-dependent form that could have been copied via emulation (a tool-use behaviour that involved making and then using a loop out of bendy material) all ape observers (gorillas, orangutans, bonobos and chimpanzees) failed to copy both the behaviour and the artefact form (Tennie et al. 2009).

Yet, the original general claim for the requirement for action copying still largely stands for a sub-part of cumulative culture: namely for those copying-dependent forms that are entirely based on actions, such as certain dance and ritual action-patterns (Tennie et al. 2012; Tennie and van Schaik 2020). Yet even here, alternative copying mechanisms are also possible, in particular instructions via language (though language itself depends on form copying), or molding/shaping of action forms (a copying variant of teaching). However, even non-copying variants of social learning can, at least potentially, lead to effects that are dependent on social learning—e.g. the know-*where* of a very well hidden source of water in the landscape (step-wise traditions; Tennie et al. 2009; See also Arbilly and Laland 2014; Heyes and Pearce 2015; Heyes 1994; Zuberbühler et al. 1996; Tennie et al. 2020).

There have also been claims (Marshall-Pescini and Whiten 2008) that when a behavioural form is unlikely to be shown by subjects, e.g. due to the situation being aversive, the resultant behavioural form must have been copied when it appears. For example, if an individual’s own motivation to engage in a situation (to reinnovate a behavioural form) is low, perhaps because it induces pain (say, during the feeding on nettle or thistle leaves), it has been argued that social mediation to overcome this low motivation, to the point of expressing/developing the behavioural form, equals copying of the behavioural form. A simple example demonstrates that this proposition is incorrect. Most, dogs can naturally swim (i.e., a specific form of action patterns involving paddling with their feet). Yet, some are reluctant to do so, or even to go near water. Dog owners, or other dogs, can however motivate—and with it, socially mediate—these reluctant dogs to swim (and the rest would swim if they were to fall in water). Does this mean that the dogs learned to swim by having to copy the behavioural form? Of course not. Many mechanisms can socially increase the motivation to engage with an aversive stimulus—but copying is only one of many possibilities. Hence, a mere socially mediated increase in motivation to do X does not—by itself—pinpoint copying of the form of X.

Lastly, it is worth noting that sometimes a behavioural form shown to be a latent solution (because it is reinnovated by naïve subjects) can also be expressed by a species that does not practice it in the wild. For example, while food-mining behaviour was observed originally in wild monkeys (Kawamura 1959; see also Galef 1992), it was subsequently documented also in captive, ecologically representative apes (Allritz et al. 2013). This suggests that latent solutions do not only need to be restricted to what we observe in the wild or to a particular species. Indeed, latent solutions can also appear whenever a new situation presents itself to a species, for example if a problem appears to a species capable of solving it (e.g. no bird species evolved to open milk bottles by pecking at them, yet several species can do so, without the need to copy the behavioural form; Sherry and Galef 1984, 1990). It can even be argued that all studies that have previously examined purely physical cognition—problem solving in ecologically representative subjects—could be meaningfully re-interpreted as latent solution experiments.^21^ Spontaneous performance in these tasks helps chart a species’ ZLS (and species’ overlaps in their ZLS).

## Teasing apart terminology

The use of the term “solution” within “zone of latent solutions” has been questioned. Moore (2013) criticised the term for being vague. For example, Moore suggested that some might argue that chimpanzees’ grooming handclasp behaviour (where two chimpanzees raise and touch arms whilst grooming), which was categorized as a latent solution in Tennie et al. (2009), should not be considered a solution as it is not clearly ‘solving’ any problems. Yet it seems impossible to infer any, or all, potential benefits (solutions) that a particular behavioural form might offer. Regarding the grooming handclasp behaviour, perhaps it is a solution to move unwanted/unnecessary arms out of the way during grooming bouts. Indeed, in general it is difficult to exclude “solution aspects” of even seemingly arbitrary behaviours. For example, some orangutans have been observed using leaves whilst producing “kiss-squeak” noises (van Schaik et al. 2003). While the use of leaves in this behaviour may at first appear to be highly arbitrary, the addition of the leaves changes the sound properties of the vocaliser—making the individual presumably appear bigger than they actually are (Hardus et al. 2011). This behaviour, therefore, may be called a ‘solution’ after all (namely a solution to the need to appear big despite lacking mass).

Furthermore, regarding the name of the hypothesis, Moore (2013) argues that the original conception of the ZLS (Tennie et al. 2009) included individually learned behaviours, and that this appears to be at odds with the formulation that the behaviour is “latent”. In Tennie et al. (2009), the term “latent” implied that every behavioural (or artefact) form within the ZLS could, in principle, arise in a similar form (see Fig. 1b) without form-copying access to specific cultural histories (i.e. could (re-)appear despite poverty of observed form stimuli). Individual learning is one way in which this can happen. More generally, a behavioural form is “latent” in any interplay between genetic setup and environment (excluding form-copying). Therefore, to be very clear: the ZLS includes everything within this interplay, from what may be called “hardwired” behaviours (e.g. yawns) to those at the very edge of individual learning/innovativeness of a species (e.g. nut-cracking in apes, which can be reinnovated (see above) but relatively rarely). In other words, the ZLS ranges from what some may still call instinct (though we prefer not to use this term; Bandini and Tennie 2018) to complex physical cognition—so long as none of the resulting forms are copying-dependent. “Solution” relates to a repeatable form across individuals (often, not always, a literal solution to a problem), and “latent” relates to a lack of need for form-copying to produce the form (and the whole ZLS term also has a historical aspect to it, see next section). Note again that non-copying variants of social learning—minimal culture—can also often play a role alongside the gene-environment (and ontogeny) interplay. Usually this role will be in regulating frequencies of reinnovations, but at times minimal culture might lead to so-called grey zones of cumulative culture too—where socially mediated, yet still individually expressed, forms may increase likelihoods of further individually expressed forms down the line (Tennie et al. 2020).

## ZLS contrasted with Vygotsky’s zone of proximal development

The ZLS hypothesis—and certainly its name—was in part inspired by Vygotsky’s approach to children’s learning abilities (e.g., Vygotsky 1978). It is, therefore a risk that the ZLS hypothesis may be seen as a reformulation of Vygotsky’s (1978) zone of proximal development (ZPD; the zone, or rather the bundle of mechanisms, that increases human skills through cultural learning). However, this is not the case. Reindl et al. (2018) explain that the ZLS should be regarded as the largely phylogenetically derived “baseline” version of a different one of Vygotsky’s zones: the zone of actual development (ZAD). This ZAD can be best described as the current skill level reached by a human.^22^ Note that the addition of a (baseline) ZLS contrasts with Vygotsky’s originally envisioned ZAD concept. Vygotsky, perhaps because he worked exclusively on human children and not apes, dismissed the necessity of a ZLS-like “baseline zone” for humans (a form-copying-free baseline, that is). Yet, there is empirical evidence that this zone also exists for humans: Reindl et al. (2016) provide data on some of the tool-use behavioural forms that are within human children’s ZLS that do not seem to require copying (as they can be reinnovated by task-naïve children (note that the validity of this conclusion was increased by taking tasks from different ape species)). This finding has now been replicated and extended cross-culturally, which further demonstrates that it does not depend on specific cultural—including form—backgrounds (Neldner et al. 2020). Thus, the ZLS hypothesis completes Vygotsky’s original ZAD and ZPD concepts by providing a missing third zone: the ZLS (as the largely phylogenetically-based and form-copying-independent baseline of the ZAD).

In the end, much depends on the innovative ability of a given species.^23^ To simplify a little, in species with limited innovative ability, form-copying is hardly even necessary as form innovations can be made by naïve individuals exposed at the right time to similar conditions cued through social mediation. Yet, if a species’ innovative ability is more variable and complex, chances are that an individual will make cumulative form innovations (which may merely build on others the same individual made before; Tennie et al. 2020). In these cases, form-copying could become more useful for others to efficiently acquire cumulative innovations directly, allowing for a potential feed-forward coevolution between innovative abilities and form-copying. In such a species, the ZLS would turn ontogenetically into the ZAD, and then into the ZPD. So far, such variability, major innovations and the accompanying processes are known for humans only—unless some animal song can be taken into account—although how (cf. Pradhan et al. 2012; Laland 2017) or when (Tennie et al. 2016, 2017) our ancestors arrived at this point remains debated. However, some species may be intermediate and show grey zones of cumulative culture (and this may be true also for apes; Tennie et al. 2020—see also Price et al. 2009), but even then their expressed conditional forms may remain stuck in individuals due to poor form-copying ability, at least when they are based on actions.^24^

## Learned versus innate behaviours

Gruber et al. (2012) incorrectly re-described the ZLS hypothesis as the “ontogenetic flexibility hypothesis,” according to which population differences in chimpanzees develop entirely in reaction to environmental variations that might exist between the populations. This is incorrect. Just like the genetic level, the environment is an integral (though again, not the only) aspect of the ZLS hypothesis and has been from the original formulation (Tennie et al. 2009). Similarly, Sterelny (2012) claimed that the original ZLS hypothesis of 2009 does not allow for adaptive social learning of any sort. However, as already explained in an endnote of Tennie et al. (2009), “low-fidelity” (i.e. what we here call non-copying)^25^ social learning mechanisms can result in adaptive behaviour of populations by leading to ‘step-wise’ traditions, for example via a cultural evolution of “know-*where*” (Tennie et al. 2009). Given recurrent misinterpretations, more details are needed about how the ZLS hypothesis exactly accounts for the variations in emergence as well as maintenance of population level differences (or universals) of frequencies of behavioural forms. We will attempt to further clarify this point below.

Ontogenetically, behaviours derive from a complex interplay of at least three broad factors (note that for readability reasons we will leave out in the following the additionally relevant levels of evolution): (1) the genetic endowment, (2) the physical and biotic environment, and (3) cultural influences (including in some species also form copying)—and together, these factors constitute “triple inheritance” (see below)). Moore (2013) is correct in noting that the ZLS approach does not focus on how strongly—or in exactly what way—the first two factors interact in order to produce a form. This is in large part because the categorical distinction between innate and learned is artificial and refers to the obsolete nature/nurture debate. Yet, the lack of this particular focus is intentional for another reason: the main aim of the ZLS hypothesis is to assess the *necessity* of the third factor—culture, especailly form copying variants—in explaining the form of a trait. Fortunately, individual cultural influences (e.g. know-how (form) vs. know-where (whereabouts) etc.) can be triangulated and studied (and these influences may at times even be isolated—e.g. when testing species that lack a certain factor such as action form copying). This is what makes the cultural factor principally suitable for even dichotomous experimental manipulation (demonstrations absent/present)—for example by testing target-form-naïve subjects of species that show little if any form-copying for spontaneous reinnovations of target behavioural and/or artefact forms (e.g. Bandini and Tennie 2017, 2019; Tennie and Hedwig 2009).

## Bridgeable inter-individual variation in apes—the similarity gambit

One possible interpretation of the original ZLS formulation (Tennie et al. 2009) could be that every individual of a ZLS-restricted species is, in principle, equally likely and capable of spontaneously expressing any of the forms within their species’ ZLS. To be a useful approximation, the ZLS approach does not have to depend on an entire lack of individual variation, however. As long as individual variation is sufficiently limited, and as long as socially mediated reinnovation creates additional homogenisation across individuals’ likelihood of specific forms expressed, the ZLS approach maintains explanatory power (a point conceded also by Sterelny 2020). It is therefore an empirical question how much individual variability in form-producing abilities there is in any species suspected to be ZLS restricted. As we will show below, and in contrast to the case of humans (see also section “ZLS as related to cultural epidemiology”), the variability seen in apes indeed seems sufficiently limited for the ZLS approach to generally apply. In addition, socially mediated reinnovation empirically further reduces the variability seen in form-producing powers across individual apes. We admit that future studies could change this picture—it might be found that form-copying plays a larger role in apes and that the transmission of form may create a ZPD-ZAD axis also in apes—at least sometimes (in which case a ZLS-only approach to apes would become infeasible). However, even then, it may still be that ecologically relevant apes are more similar than different in their individual innovative powers and that these powers can be further homogenised via socially mediated reinnovation. At least that remains the general picture today (see discussion below), which allows the ZLS approach to be generally applied to apes. We can call this the “similarity gambit” of the ape ZLS hypothesis.^26^ We therefore argue that the ZLS remains currently the most relevant approximated explanation of the various forms produced by apes.

There are recent findings on differences in motivation and personalities across chimpanzees (e.g., Hopper et al. 2014; Massen et al. 2013; Altschul et al. 2017). Given this, it is possible that some individuals are more likely to express a certain latent solution’s form than other conspecifics (see, for example, Watson et al. 2018). Yet, the ZLS account allows a harmonizing of latent solutions expression among connected individuals—given the right ontogenetic timing, order of latent solutions expressed, external conditions and overall internal conditions (for further discussion, see Tennie et al. 2020). Thus, even if individual A, for whatever reason^27^ is less likely to innovate trait X compared to individual B, once A encounters B performing X, individual A’s likelihood of also performing X is sufficiently increased (all this still without any form-copying variants of social learning required; mere cues suffice). Therefore, even if there is variability in the likelihood of initial innovation of latent solutions in apes, including differences in stochastic environmental conditions catalysing the original innovation, because apes often understand their own actions and can link (to a certain degree) these actions to outcomes (and remember them), this can then ‘catch-on’ on an individual level (in the original innovator), which can then lead to a cascade of socially mediated reinnovations (as in Fig. 1b). For example, in 2010, Tennie and colleagues tested naïve, captive, ecologically relevant chimpanzees with the floating peanut paradigm, in which subjects have to spit water into a vertically fixed, top-open tube until it is sufficiently full to allow for a shelled peanut to float into reach (Tennie et al. 2010). The chimpanzees were tested in two conditions: a full demonstration condition where spitting action forms were demonstrated, and a control demonstration condition, in which no spitting actions were demonstrated (instead, here, water was poured into the tube from a bottle; as a socially mediated cue). Tennie et al. (2010) found that, after these varying demonstrations, observers in both conditions used spitting actions at comparable rates to solve the task. The use of spitting actions did not differ, regardless of whether subjects had seen the form of these specific actions or not. The most parsimonious explanation of this finding is that subjects individually reinnovated the action form that was necessary (spitting) in both conditions. Note that it had been previously shown that chimpanzees can also innovate the spitting solution entirely from scratch (Hanus et al. 2011). Taken together, the copying of action forms, as well as any copying in general (see Hanus et al. 2011), proved unnecessary to reinnovate the action forms in this task in accordance with the ZLS hypothesis and in contrast to the copying hypothesis.

Modest differences between individuals in general motivation, luck and/or even skill to produce target forms can thus be bridged by non-copying social learning, even if sufficiently large inter-individual differences would eventually require social learning via copying (as in humans). In humans, relatively large inter-individual differences in form-production skills can occur but are likely due to cumulative culture of forms and/or human cognition itself (compare Vygotsky (see above); Henrich 2017; Herrmann et al. 2007; Heyes 2018; Tennie and Over 2012, section “ZLS as related to cultural epidemiology” below). Again, the question of whether this appears also in apes is an empirical question. Therefore, the variability in ape innovation likelihood and range (across the entire life span and across a range of factors) needs to be studied in more detail (see also Tennie et al. 2020). However, we would like to emphasize that even a behavioural form that appears to be close to the periphery of ape ZLS, as is chimpanzee nut-cracking (only rarely reinnovated by naive chimpanzees; e.g., Boesch 1996; Morgan and Abwe 2006; Marshall-Pescini and Whiten 2008; Ross et al. 2010), does reach population-level frequency in some wild populations. Assuming the ape ZLS hypothesis still applies in these cases (and the cases of nutcracking reinnovation would suggest this), this would effectively mean that even this particular bridge can be crossed by apes, with the crutch of non-copying variants of social learning—further support for our ape similarity gambit. Of course, again, it is also possible that evidence will eventually be found that apes cross this bridge sometimes (or perhaps in part) by copying social learning.

In a similar vein, the interpretation of small subsets of supposedly high-performing subjects in latent solutions tests as representing the entire species has been criticised by Thornton and Lukas (2012). Their concern is that “genius” performances of—in extreme cases—single individuals of a species do not necessarily mean that all members of a species will be able to perform to the same standard. However, we do not believe that this criticism applies to the outcomes of latent solution experiments, where (usually captive, but ecologically representative) apes reinnovate (usually wild-type) behavioural forms. First, the updated operationalization of the ape ZLS concept (Tennie et al. 2020) now requires a behaviour to appear in at least two independent individuals for it to be considered a latent solution (compare also Bandini and Tennie 2017).^28^ The ape ZLS hypothesis therefore still seems best able to explain multiple independent appearances of similar forms as this requires a more homogenous spread of underlying abilities than single cases would. Second, and most importantly, our interpretation of behaviours as latent solutions is not at odds with such a description of “geniuses”. The ape ZLS concept allows for non-copying social learning to facilitate the form (re-)appearing also in others—at least once one or more individuals have innovated the behaviour and can act as cueing catalysts (Tennie et al. 2009; Bandini and Tennie 2017; see Tennie et al. 2020, see also above). Thus, our focus is simply different: whilst we are interested in the necessity of copying to explain certain forms (by testing if the same form appears repeatedly in at least two naïve, form-isolated cases in ecologically relevant apes, and by testing if such apes are even able to copy copying-dependent forms), Thornton and Lukas’ focus is on the variability of cognitive abilities across members of a species. This variability is most likely fact, but, importantly, it is probably less pronounced in apes than in humans and bridgeable by socially mediated reinnovation (according to our ape similarity gambit). This renders feasible the ZLS account for ape forms, which also provides an explanation for how even these differences may be further levelled by non-copying social learning—socially mediated reinnovation—to harmonize selections of forms (at least within affected populations). Overall, then, the similarity gambit of the ape ZLS hypothesis seems justified, at least in currently known cases, and thus, overall, the ZLS approach has sufficient explanatory power in the case of apes to be the preferable null hypothesis.

## Is the latent solution test methodology valid?

Latent solution experiments represent one main way to assess predictions of the ZLS hypothesis. They test for spontaneous reinnovation of target forms in naïve, ecologically representative ape subjects—which should after all only occur if the ZLS view is correct. These subjects (who have never seen^29^ or been trained in the target and related forms before as far as keepers in the testing institution are aware^30^ and as far as the researchers know) are provided with all the materials of the target behaviour, but no demonstrations or social information on the actions or results involved in the form (e.g. if the target form was nut-cracking, the subject would merely be provided with nuts, hard surfaces and stones/wooden ‘hammers’). If the naïve subject spontaneously performs the form of wild-type nut-cracking in this “poverty of the stimulus” condition (or baseline; compare also Heyes 2018), then it can logically be considered to be a latent solution, as its form (the know-how) does not require behavioural form copying (Tennie et al. 2009; for more details and an update on this method see Bandini et al. 2020).

Note that this methodology does not make any predictions as to the latency until individual form reinnovation. Young chimpanzees in the wild may take years to show nut-cracking forms^31^ (Biro et al. 2006), but this fact alone does not provide any information on whether the behavioural form of nut-cracking is a latent solution or not. Again, the fact that nut-cracking has appeared in spatially-distant wild populations, and therefore most likely re-emerged independently (i.e., Morgan and Abwe 2006) suggests that this behavioural form may be a latent solution, regardless of how long the new generation of chimpanzees requires before they acquire the full behavioural form.

Although this is a relatively simple method (see Bandini et al. 2020 for extensions) that allows for clear interpretations of the results, some researchers have questioned the validity of the latent solution method (see, for example, the published reviews of Bandini and Tennie 2017; Mesoudi and Thornton 2018; Haidle and Schlaudt 2020). It has been argued that the latent solution methodology involves a condensed presentation of the required raw materials to naïve subjects. In short, the criticism is that these ‘ideal circumstances’ created in latent solution tests, in which all the necessary materials are provided to the subject, do not adequately recreate the “unlikely” innovation conditions encountered in the wild. However, the aim of the latent solution test, similarly to some of the earlier classic tests on behaviour in ethology (e.g. Köhler 1925; Sherry and Galef 1984), is to determine if it is *at all possible* for a form-naïve member of a species to spontaneously (re-)produce the form of a wild-type behavioural form that supposedly requires copying to learn (Bandini and Tennie 2017; Bandini et al. 2020). This conclusion should not be problematic. As laid out by Tennie et al. (2009), the concrete details of the situation in which the original innovator(s) in each wild population found themselves in will vary stochastically (see also Bandini et al. 2020). Therefore, it is unproblematic if the situation created in latent solution tests recreates similar situations, i.e. as those sometimes faced by chance by wild individuals. These situations might present themselves rarely in the wild (which is fine), but when they do, they may facilitate the emergence of the latent solution in the original innovator(s) *in the same way as they do in latent solution experiments*.^32^ The test may also be started with a less condensed distribution of the necessary raw materials. Sometimes a latent solution test can even be run at places where the necessary raw material has already been available to subjects prior to the test (e.g. a potential source of sticks in a bush in an enclosure; Bandini and Tennie 2019) and so, additional enhancements due to to the introduction of such material can be avoided in such cases (see Bandini et al. 2020 for an updated and detailed methodology of latent solution and follow-up tests).

Even more importantly, and even in the wild, once others observe or interact with these original innovators performing the behaviour (including the products of their behaviours), these observers are effectively socially placed in the same situation as the subjects tested with the latent solution method, in that the materials are then necessarily presented in an enhanced and “concentrated” and therefore salient way—they then act as relatively specific cues for the individual reinnovation of others (as in Fig. 1b). Thus, even when raw material is provided in a somewhat salient, enhanced way, latent solution tests can reproduce the context that *re*innovators encounter in the wild. By not introducing other, irrelevant, materials to the task, and only providing them with those required to solve the problem at hand, we increase the chance that subjects will be interested and motivated in the materials we do provide.^33^ In both cases we can therefore expect an increase in the likelihood/frequency of individual reinnovations of forms (which is, after all, a large part of the explanatory power of the ZLS account). Crucially, the form of the behaviour is still not demonstrated, and thus must derive individually. Furthermore, a new, updated latent solutions methodology (Bandini and Tennie 2018; Bandini et al. 2020) now allows for the role and necessity of different variants of social learning (both copying and non-copying) in the acquisition of a behaviour to be identified sequentially or in parallel in additional test conditions. Therefore, to the best of our knowledge, the updated testing methodology provides an appropriate and valid experimental procedure to test for the required individual and social learning mechanisms behind the acquisition of a target form by form-naïve, ecologically relevant subjects (contra Schofield et al. 2018).

Lastly, Mesoudi and Thornton (2018) criticised the ZLS account on two additional fronts. First, they argue that it is impossible to test fully naïve humans in latent-solution-type tests (compare also Haidle and Schlaudt 2020) and, second, they suggest that the baselines provided in latent solution tests are too short—typically only lasting a couple of hours, rather than the target ‘lifetime’ occurrence probability (*ibid*). The problem is well-known, but the question can be triangulated in several ways. With regards to the first point, the latent solution testing methodology focuses on target forms for which one has reasons to believe that they are unlikely to have been, directly or indirectly (see section “ZLS as related to cultural epidemiology”) copied by test subjects. For apes and humans alike, we argue that it is possible to find subjects that have not observed the target form directly, as long as the target form does not occur in the subject population at all, including (in the human case) access via modern media.^34^ For apes, we can also (largely) exclude indirect effects (of perhaps related forms copied in the past)—simply because ape form copying skills are very much limited (and likely even absent in the action form copying domain). Another related way to test latent solutions is to test younger individuals as these (everything else equal) are less likely to have already seen target or other forms than older individuals—and the validity of this approach can be even further increased when a multitude of target forms is tested and when, additionally, target forms are taken from a different species (e.g. see the approach used by Reindl et al. 2016). Validity is again further increased when the specifics of the surrounding cultural (including its contained forms) and environmental settings does not change the results in substantial ways (see the approach used by Neldner et al. 2020).^35^ Finally, for many modern human copying-dependent forms, history provides ample valid natural latent solution experiments. For example, we know that smartphones are copying-dependent forms as they did not appear in hundreds of thousands of years across millions of subjects—and we can meaningfully posit that this was because these people did not have access to the cumulated cultural forms that are behind smartphones (of course, other factors need to be present too; Tennie et al. 2009). We can infer that these people were fully naïve to the target form and related, underlying forms (see also section “ZLS as related to cultural epidemiology”). Hence, we can indeed know that the production of the form details^36^ underlying smartphones (and planes, and modern dance and music plays etc.) is not due to a latent solution (compare also the “lost explorer” natural experiments as described, e.g. by Boyd et al. 2013).

With regards to the length of latent solution tests, currently, there is indeed no standard yet on how long these conditions should be (but for a start, as a rule of thumb, Bandini et al. 2020 suggest such tests be at least double the time length of social learning conditions). Furthermore, when designing a latent solution experiment, one must also take into account the levels of motivation and fatigue of the participants, which might decrease and increase respectively along with the length of the test (therefore potentially reducing the utility of longer testing conditions). Anecdotally, it seems to one of us (CT) that ape testing does suffer from this particular problem; if true, this would render the first testing sessions more valuable than later ones (which can introduce carry-over effects to later conditions). Yet, more importantly, Mesoudi and Thornton’s (2018) critique is empirically largely moot: previous latent solution studies have demonstrated that target forms were often reinnovated in concrete examples of such tests, sometimes very fast (Allritz et al. 2013; Bandini and Tennie 2017, 2019; Menzel et al. 2013; Neadle et al. 2017; Reindl et al. 2016; Tennie et al. 2008).

## There can be multiple latent solutions within a single domain

A particular problem or situation (including a social situation) does not necessarily only have a single possible latent solution (Tennie 2019a). For example, the chimpanzee behaviour of ant-dipping, which involves using sticks to retrieve ants from their ant holes, is expressed in several form-distinct ways: direct mouthing with a short stick; pull through with hands with a long stick; or pull through with mouth with a long stick (see Boesch 1996 for descriptions). Because each of these three reported methods for ant dipping may represent a latent solution, the test case would then again be if *all* of these forms emerged in naïve subjects in baseline latent solution tests.^37^ This is related to the finding that, in two-target tasks, there is often the illusion that forms are being copied (e.g. push door left vs. push door right), yet copying—especially action form copying—is not required if all form variants can be reinnovated by naïve, ecologically relevant subjects (Bandini and Tennie 2017, 2019; Tennie et al. 2020; see also Fig. 1b). Whether a population then preferentially choses one form X over other, possible forms (Y, Z etc.) will depend, as always, on specifics of triple inheritance—which can include potential specific socially provided cues in the sense of Fig. 1b but without the necessity to assume form copying (though form copying may of course, sometimes happen—an empirical question).

## The role of social learning biases

Social learning might be biased in the sense of, e.g. *when* to use social learning, or *who* to be influenced by. The original ZLS hypothesis formulation did not discuss these social learning biases, which are also known as social learning strategies and transmission biases^38^ (e.g. Laland 2004; Rendell et al. 2011; Richerson and Boyd 2005; Kendal et al. 2018). This was because of constraints on length, but also because not much empirical data on social learning biases in primate social learning was available at the time. Whilst more is known now, the results are mixed (e.g. van de Waal et al. 2012; Kendal et al. 2015; Vale et al. 2017; Watson et al. 2017; Botting et al. 2018). Furthermore, and critically, these studies did not differentiate between the copying of traits versus mere increases in frequencies via socially mediated reinnovation (Bandini and Tennie 2017). While copying-dependent forms can evolve even via random, unbiased copying (drift due to the associated error of copying), transmission biases could be very helpful in providing a direction for the copying. Hidden in plain sight, for example, transmission biases underlie the original formulation of the ratchet effect (Tomasello et al. 1993; Tennie et al. 2009) in which better variants are supposed to be preferentially copied (i.e. a copy-if-better social learning bias). However, all this is not to say that social learning biases would not also meaningfully affect the outcome of non-copying social learning (i.e., biases for the resulting frequencies of latent solutions). In fact, social learning biases are—at least principally—mute on the social learning mechanism variant that they may affect. Principally, there can be copy-if-better, or conformity biases, or any other social learning bias even in non-form-copying social learning mechanism (e.g. in local enhancement; compare step-wise traditions; Tennie et al. 2009). Therefore, if social learning biases are present (and indeed they are likely to be present in many species; including apes), then we can expect them for example to bias socially mediated reinnovations—e.g. they may affect whether and/or how fast specific latent solutions will increase or decrease in frequency (compare data in Nishida et al. 2009; Leca et al. 2010).

## Species evolve and so do their ZLS

Another potential misunderstanding of the ZLS hypothesis is the idea that a species’ ZLS is immutable. This is incorrect. Instead, a species’ ZLS no doubt can change during the species’ evolution.^39^ Depending on the force and time patterns of past evolutionary drivers (including feedback generated by minimal culture), some latent solutions may have even become developmentally more canalized (leading to less variability in expression and/or increased likelihood or speed of expression). Even where they are not, some similarities in cognition are owed to shared recent ancestry, and therefore we should not be surprised to see an overlap in the ZLS of species that share recent common ancestry.^40^ We also can expect to see convergent evolution of latent solutions in species that do not have a proximal shared ancestry but have instead faced similar evolutionary pressures and/or trajectories (a possible candidate case here might be nut-cracking across primate species; found currently in chimpanzees; e.g., Boesch and Boesch 1991; in capuchins; Ottoni and Mannu 2001 and in long-tailed macaques; Luncz et al. 2017). Note, however, that latent solutions are not restricted to adaptations; they can also be exaptations (Gould and Lewontin 1979)^41^ or any mix between adaptations and exaptations. Problem-solving cognition could even be re-described in this context as an exaptation generating machinery (compare also Tennie 2019a).

This leads to an interesting conclusion: whether a form is copying-dependent or not can therefore also depend on when we look at the lineage. For example, Levallois-style stone tools may have been a copying-dependent form for earlier Oldowan-making hominins, but might not have been copying dependent for the (later) hominins that actually made Levallois stone tools.^42^ Potential evidence for the latter comes from proposals that there have been multiple independent reinnovations of Levallois production techniques (at least when there was a (ZLS?) background of the Acheulean; White and Ashton 2003).^43^ Similar reasoning applies to the Acheulean versus the Oldowan and potentially other early stone tool forms (compare also Tennie et al. 2016, 2017).

## The larger theoretical framework of the ZLS hypothesis—triple inheritance

We envision the ZLS hypothesis to be inside a general framework in which multiple factors may be inherited, in sometimes very different ways, and in which these inherited factors interact with each other over time. While the full story will necessarily be more complex than a broad framework, there would seem to be three main categories of inheritance: genes, culture and the environment. These categories could be split up even further (e.g. form-copying or not), and while some other important factors do not fall neatly into these categories (e.g. epigenetics; developmental constraints, ontogeny in general etc.), together these three major categories cover sufficient ground to be useful.

All three of these interacting factors are, generally speaking, heritable, and this holds true even for the physical environment. That is, our approach is located in a “gene-culture-environment co-evolutionary framework”; i.e. adding environment to the usual “dual inheritance” framework (e.g. Boyd et al. 2013). Consequently, this three-linked-column approach has been dubbed triple inheritance theory (see Laland et al. 2000; Odling-Smee and Laland 2009). Triple inheritance allows for the examination of the relative importance (and timing) of each of the constituting inherited factors for any concrete phenomenon. Answers can meaningfully differ between phenomena. There may be cases—in humans as well as in non-humans—where the environmental level contributes more heavily to a concrete phenomenon than the other levels. In yet other cases, it may be the genetic level. In others, culture (the main focus of the ZLS hypothesis). Sometimes each factor may play an equally important role. The major usefulness of the triple inheritance approach lies in that it does not prioritise any of its factors over others per se (unlike, e.g. evolutionary psychology, human behavioural ecology, human socio-biology or cultural evolution theory) and in that it is more succinct, yet in our view not less precise, than “dual inheritance plus environmental niche construction”.

## ZLS versus evolutionary psychology

Evolutionary psychology distinguishes between evoked culture and transmitted culture (Tooby and Cosmides 1992; Gangestad et al. 2006). Transmitted culture has some overlap with triple inheritance, but is much less developed and, most importantly, seemingly committed a priori to a nativist view (perhaps unsurprisingly). In particular, it regards human social learning mechanisms (and social learning biases) as natural adaptations (Gangestad et al. 2006). Our perspective is less predetermined. Within triple inheritance, broadly understood, it is an empirical question which factors play a role in what way to produce the social learning mechanisms and biases underlying culture. Indeed, there is mounting empirical evidence that some of these mechanisms and biases are largely culturally transmitted themselves, in the case of modern humans (see Heyes 2018). While the ZLS hypothesis need not commit to how these mechanisms come about in humans, note that the evidence on ape form copying (weak at best in ecologically relevant apes, but clearly present in human-enculturated apes) is largely in line with Heyes’ views (Tennie 2019b).^44^

The second variant of culture within the evolutionary psychology framework is evoked culture. It can be described roughly as different environments producing different human behaviour patterns without involving any social learning at all (Tooby and Cosmides 1992). In this account, each behavioural outcome pattern is somewhat predictable and this predictability is mostly due, again, to natural adaptations (Gangestad et al. 2006). Although the evoked culture and the ZLS concepts overlap to a small degree, there are also important differences. First, the ZLS hypothesis is foremost interested in explaining non-human culture. Second, while the ZLS does not exclude adaptations (see above), it does not restrict itself to adaptations. Third, the ZLS hypothesis splits up social learning mechanisms, and takes seriously the differential effects of these different social learning mechanisms. On the broadest level, the ZLS hypothesis inserts a wedge in between the two culture variants of evolutionary psychologists—by describing (minimal) culture not based on copying of form. That is, the ZLS largely fills a middle ground that is missing in evolutionary psychology.

## ZLS as related to cultural epidemiology

Scott-Phillips (2017) was the first to explicitly connect cultural epidemiology (CE; Sperber 1996)^45^ and the ZLS hypothesis (ZLS). Scott-Phillips noted that while CE provided insights for human cultural phenomena, the ZLS hypothesis explored cultural phenomena in nonhuman animals. Specifically, Scott-Phillips (2017) concluded that the ZLS hypothesis “attempts to meet the important challenge of identifying which cultural items are easily re-producible for a given species”.^46^ Here, we solidify, summarise, and, sometimes idiosyncratically, extend CE’s position (but in all cases with goodwill) to help provide greater clarity between the links and lack thereof between CE and the ZLS hypothesis. As before, we will focus on explanations of forms.

Perhaps given its focus on humans, one of the strongest and (to many) most surprising claims of CE is that copying of form itself has a relatively small (and/or rare) role to play in explaining human form phenomena (e.g. Sperber 1996, 2000; Morin 2015). According to our reading of CE, it is (mostly) the individual (or rather, its summed properties, see below) that creates these forms (compare Acerbi and Mesoudi 2015). Here, the ZLS would indeed appear to be similar to CE—the ZLS, too, links form (at times even exclusively) to this individual level (mostly in apes, but also in many other animals). To clarify further, CE promotes a former’s eye view to explain human form phenomena (but note that because using the term “former” to characterize CE would lead to semantic confusion we shall instead refer to CE as promoting a *constructor*’s eye view). Additionally, CE holds that the constructor’s eye view is overall the more appropriate view than the perhaps more usually taken form’s eye view (compare Sperber 2000). This alternative form’s eye view holds instead that forms are passed on (and can thus evolve) via copying—more specifically, that they require copying (though selection plays a role, too; Acerbi and Mesoudi 2015). Here, copying both allows and fuels the so-called ratchet effect underlying form cumulation (e.g. Tomasello 1999).

Yet it is still unclear how a world consisting mainly of constructors could produce and maintain the vastly different forms we see in modern humans (and in such short time). The current answer provided by CE,^47^ at least in our reading, lies in linking form variability not to forms (and form copying), but to the underlying variability of constructors. Different forms would then derive from, in essence, different constructors (compare individual “reconstructing”’; Sperber and Claidière 2008).^48^ Given this premise, the CE approach must therefore—at minimum—be able to show that (and how) constructors come to differ in ways that shadow modern human form phenomena. Three main factors have been proposed as potentially differentiating constructors: 1) cultural factors (Morin 2015, Sperber 2000, Claidière and Sperber 2007; Buskell 2017), 2) genetic factors (e.g. Sperber 1996, 2000, Sperber and Hirschfeld 2004, Claidière and Sperber 2007); and/or 3) environmental factors (e.g. Sperber 1996, Morin 2015, Scott-Phillips et al. 2018; compare also Acerbi and Mesoudi 2015).^49^ Indeed, logically, CE may as well go one step further (compare Sperber 1996; Buskell 2017), by including differentiations on the “output” side too. Here, the resulting constructors’ tendencies may not only influence cultural forms, but also genetic forms (e.g. gene editing; artificial selection) and environmental forms (e.g. changing landscapes; artefact forms). Overall, we may thus envision populations of constructors formed by three factors *and* forming three factors that again form individual constructors etc. The combined effect then can be differentiated evolving and complex systems, even on the population and sub-population level. Such a complex, multi-factorial, evolving, yet potentially patterned, system could indeed—at least in theory, but perhaps not in current practice^50^ explain human form phenomena, including the observed differences and explosions in form,^51^ alongside accurately tracing populations and sub-populations. The constructor’s eye view (CE) and the form’s eye view could therefore in principle lead to equifinal form expectations from the micro- to the macro-level. The ZLS hypothesis likewise maintains that the forms shown by apes (and many other animals) are best explained by factors other than form-copying, i.e. by a constructor’s eye view of sorts (see above). More precisely, both CE and ZLS argue that specifics of output form may be independent of specifics of “input form”.^52^ However, again, the viewpoints diverge when it comes to explaining human phenomena (see, e.g. Tennie et al. 2009, for a form’s eye view on human form phenomena instead), and the accounts also differ in other respects, which we turn to below.

We can safely—and empirically—assume that human form phenomena are not always pure copies *and* not always derived entirely from individual constructors (Acerbi and Mesoudi 2015). In this way, both viewpoints would seem to have merit. The three main underlying factors for any current view of human evolution seem to have converged on the co-evolution of genes, environment and culture (triple inheritance). Given that our interest here lies in explaining forms produced by modern humans (which have exploded both in number and specifics in short time frames), we can simplify our approach substantially as follows. While all three factors of triple inheritance clearly play a role (even if often in merely constraining form, one of the foci of the ZLS), we need to first ask which of these three (genes, environment or culture) has the highest explanatory power for accounting for the form explosion and maintenance seen in modern humans. Genetics cannot explain this well—for the simple reason that there are no meaningful systematic genetic differences between modern human populations that could be tied to it. Environment may at first seem a better possibility in explaining the different forms, but not only did European “lost explorers” repeatedly fail to re-produce the required forms in specific environments (Boyd et al. 2013) but even life-long exposure to certain environments alone will largely fail (at least, we can safely assume so) to elicit associated forms (e.g. one does not learn a specific dance form merely by spending extended time on its associated dance floor). We can conclude from all this that the human form phenomena do not essentially depend on the genetic level and they do not seem to depend on the environmental level either (at least not to a large enough degree). As we describe above, forms can be culturally transmitted via copying (at least when a species is capable of such copying, for whatever reason). Given that we only have culture left as an explanation, and given reliable copying by modern humans (e.g. see Tomasello 1999), it follows that human form phenomena most likely depend foremost on the cultural level.^53^ Overall then, we shall focus our efforts below on what seems to be the main driver of modern human form phenomena: culture.

CE promotes the idea that copying is often an illusion (Sperber 2000; Morin 2015). Indeed, technically, anything one may call “a copy” can be called a reconstruction. If one were to take this technical position, then even the genetic code would not be considered a copy but would be reconstructed instead (which, technically, would be true). Yet the specific constructor of the genetic code (DNA polymerase) is, for current purposes, input-and-output-form-neutral. The only way, therefore, for the input and the output to match is to actively make it match by copying it. To the extent that we can talk about copying at all, the genetic code is copied (note, CE does not deny the existence of copying; Sperber 2000). A second view of the problem, and one more in accordance with CE main critique, would be that even what might appear to be attempted copies of form are often too influenced by mental inferences made by the new constructor towards the old constructor to count as copies (Sperber 2000). However, not only is this situation rather similar to supposedly copying-*aiding* joint attention/joint intention sensu Tomasello and colleagues,^54^ but detailed, namely step-wise goal inferences can help along copying of associated artefact and/or behavioural forms—in which case mental inferences can actually heavily increase form copying fidelity instead (Acerbi and Tennie 2016). More generally, whether or not form-copies are made, and how often this happens, cannot be answered theoretically. How much the form’s eye view matters therefore remains an empirical issue (compare Acerbi and Mesoudi 2015).

How can we characterize copying? Here, we define “*direct* copying of form” as a *measurable matched resemblance*^55^ of the specific form of input A to the specific form of output Z, where this matched resemblance between A and B is and must be, at least in part, *causally due* to the specific form of the input (compare Sperber 2000). Given our focus here, a causally dependent match in behavioural or artefact form is therefore required.^56^ For our purposes, this does not have to happen immediately, or does not depend on there being only one single instance of a specific form as input (see also Morin 2016). Note that there are however cases where more than one input form (A to n) may at least contribute somewhat to causally linked output Z (e.g. donut + croissant = cronut^57^)—we call this latter case indirect copying of forms (or recombination, in Muthukrishna and Henrich’s 2016 terminology). Below we will first explore the case of direct copying, before turning to indirect copying (see also Neldner et al. 2020 for a similar differentiation).

Recall that the conclusion for copying might be due to an illusion. The very idea of a mere illusion of copying sounds strange at first, yet there are real life examples. As the ZLS reinnovation data shows, this situation is in fact a typical (if not the sole) situation for wild ape forms (an illusion which led so many to erroneously believe that copying has to take place). However, there are also examples in humans. Not only can young children spontaneously reinnovate ape behavioural forms of tool use (Reindl et al. 2016; Neldner et al. 2020), but various form phenomena reliably occur and stabilise at the end of so-called transmission experiments in humans without ever having been inputted at start (e.g. from simple drawn data patterns to lab-based language structures (compare Kirby et al. 2008)). To some degree this even extends to real life transmission—at least some aspects of alphabet forms may derive this way (Morin 2016).^58^ Yet, even without such transmission chains, there are rare examples that best fit a constructor’s eye view even in the human case. Sperber (2000) mentions contagious laughter (form not copied), to which yawning, smiling etc. might be reasonably added. CE can work, even in real life.

Yet, taking the general possibility of CE’s position seriously (due to some specific cases) does not answer the question of whether CE can fulfil its additional aim (e.g. Morin 2015; Sperber 2000) of explaining modern human form phenomena more appropriately or thoroughly than a predominantly form’s eye view. In order to fulfil this additional premise, CE would need to explain most examples or most aspects of human form phenomena. However, in line with others’ views, we propose that the alternative, form’s eye, view better explains modern human technology (see also Boyd et al. 2013, Sterelny 2017 and Acerbi and Mesoudi 2015). Without copying access to specific target forms as direct input, many technological solutions cannot be reinnovated. This proves true even when the constructors’ lives depends on such reinnovation, as the tragic case of “lost explorers” so vividly showcases (e.g. Boyd et al. 2013). However, as Acerbi and Mesoudi (2015) caution (and show), the applicability of CE may instead simply be better suited to other, non-technical domains. However, the general conclusion for the appropriateness of the form’s eye view often also extends to the social domain. For example, virtually every song and book are in this way copying-dependent-forms—namely whenever they re-appear (every book copy, every reading, every time a song is played etc.). It would be equally impossible to individually reinnovate the opaque food processing technique the lost explorers so direly lacked, as it is to individually rewrite (without copying access, in the past or present) Shakespeare’s Romeo and Juliet in its original specific form (compare Acerbi and Mesoudi 2015). The impossibility of these tasks (without copying access) would be beyond predicting tomorrow’s lottery numbers. All cases of such copying-dependent forms therefore appear to favour a predominantly form’s eye view. By sheer number of applicable cases, a form’s eye view would therefore seem to explain human form phenomena better than a predominantly constructor’s eye view could.

Direct copying of form is therefore required for a very large number of human form phenomena. CE might object that an alternative view could explain modern human form phenomena—not via direct copying, but via *indirect cultural influences*. This view seems untenable for the millions (or even billions) of form phenomena we relate to above. The reason is, that in order for this alternative indirect cultural view to succeed, constructors placed in such cultures would need to prove able to in essence recreate very similar forms from scratch without the specific form as input. Again, this is an impossible task. And even if we greatly reduce granularity (following Acerbi and Mesoudi 2015) even moderately similar forms might not regularly re-emerge in such subjects. CE might counter with several objections to this characterisation, which we will look at below.

First, proponents of CE might object by claiming that the specifics of the input form do not matter as the constructors must only be able to construct most of the output form, but not all. As the case of the lost explorers show, at the very least some input must be required that is associated with the specific input in question (Boyd et al. 2013; Acerbi and Mesoudi 2015). In other words, CE might claim the input is still required^59^ but that either the input’s specific form or something associated with it may merely serve as a recognisably “link” or cue to similar, and still specific, output forms Z but where Z is within or near reach of the constructors.^60^ Logically, in such cases, the constructor would have to individually reconstruct the vast majority of all specific parts of the specific input form, including their specific relative position. While this may occasionally even be the case also in modern humans (e.g. yawning forms, but also some basic tool use; Reindl et al. 2016; Neldner et al. 2020), the combined likelihood of this happening for millions of specific human form phenomena (e.g. all books ever written, all machines ever built, all songs ever sung) can likely be ignored.

Second, as we have already established, the form phenomena we are interested in here cannot rely on the genetic level and/or the environmental level. These phenomena must therefore depend in some way on the cultural level. This may mean, however, that it depends on general cultural background or, more specifically, it might depend on the indirect copying of related behavioural and/or artefact forms in the past. In other words, if a given constructor A has Z in their repertoire (where Z is a modern human form phenomenon) this may causally be so because A has copied necessary and related forms (e.g. X and Y) in the past. A general effect of cultural background, especially in the case of humans, is now widely accepted. In other words, the view that constructors influence culture, and culture influences constructors has become widespread. For example, Muthukrishna and Henrich (2016) point out that the seemingly independent development of the idea (or, perhaps best described as the “idea form”) of natural selection by Wallace and Darwin was dependent on culture. Yet, the form of the idea was not directly copied between these two men. Instead, the development of this idea might have depended on what we here called indirect copying of form. In particular, both instances of the idea expression relied on the specific cultural background that was available to both Wallace and Darwin (see also Bowler 2013). CE might object that it is not clear whether the cultural background in the case of Wallace and Darwin’s idea formulation required form copies (memorized) via past copying events. In other words, CE might or might not concede that a general cultural background was necessary, but not that this cultural background is best characterized as consisting of form copies. Yet, given the seemingly grounded fact (see above) that there exist a very large number of environment- and genetic-independent forms in humans that very much depend on direct form copying (copying-dependent forms), and given the very different and constrained forms seen in other apes (who clearly show much less form copying, and seemingly no copying of copying-dependent form), we think that we can wager a claim of the likely importance of form copying also beyond direct copying and for human cultural backgrounds in general. We believe that it is direct *and* indirect form copying that provides an important cultural background that underlies much of modern human form phenomena. Admittedly, currently this is merely an informed guess. Yet, if this view were correct, both instances of the “idea form” of natural selection would have been dependent on indirect form copying of specific aspects of a shared cultural background—and usually remain dependent on *direct* copying from these sources and following sources today.^61^ Moreover, if this view were correct, then the majority of modern human form phenomena should be best seen as direct and/or indirectly copying-dependent-forms (compare also Tennie et al. 2020). Given all the above (and again, the contrasting case of other apes), we argue that even such a heavy reliance on the form’s eye view, in a multidirectional, mixing view, reflects the modern human situation more accurately than a predominantly constructor’s eye view.^62^ We therefore reject CE’s claim of superiority (see e.g. Sperber 2000). Currently at least, the multidirectional form copying view must remain a hypothesis, not least because the constituting forms—and especially the indirectly copied, and merely memorized forms—remain difficult to detect and/or quantify. We are not claiming that the human system is well enough understood—or that the various pathways of culture are as of yet sufficiently traceable (compare Nettle 2018), to test this hypothesis. Yet we can, for now, consider this informed possibility.^63^

In sum, there are superficial similarities between the ZLS and the CE. In both accounts, there is a de-emphasis of direct form copying and an acknowledgement of the possible influence of genetic and environmental factors on forms and their frequencies. However, dissimilarities prevail. The ZLS takes seriously the various downstream effects of different social learning mechanisms and actively emphasises the important direct and indirect effects of form-copying, but only for cases where this type of learning exists (e.g. in modern humans; Tennie et al. 2016, 2017; but much less so for apes, if at all). CE also places heavy emphasis on mentalizing—but as the powers of mentalizing of non-humans are likely low (and in addition, unlikely to differ much in ecologically relevant apes), the ZLS—with its focus on apes—does not currently factor in such mentalizing effects. Lastly, the empirical likelihood (see above) of modern human dependence on both direct and indirect form-copying weighs uncomfortably against the CE’s claim of being a superior explanation despite it de-emphasizing the role of form copying (e.g. Sperber 2000). Note that for ecologically relevant apes,^64^ the overwhelming evidence against an importance of direct form copying would also appear to automatically exclude indirect form-copying effects, which is why the ZLS approach does not currently factor in either direct or indirect form copying effects (but see Tennie et al. 2020 for more in-depth discussion of a potential grey zone of cumulative culture in apes and hominins—of possible effects of past individual reinnovations (whose likelihood is under social influence) on constructors’ future innovations).

## Conclusion

In this paper we addressed the main misunderstandings of and objections to the ZLS hypothesis and located it relative to other approaches. We hope that these issues have now been clarified, and that the explanatory power of the ZLS hypothesis in general, and in its specifically proposed ape ZLS (Tennie et al. 2009) and hominin ZLS variant (Tennie et al. 2016, 2017), can be properly evaluated. As discussed, on some level the ZLS approach is not at all at odds with a social learning hypothesis regarding some, or even a major, role of social learning in the acquisition of ape behavioural forms. However, only within the ape ZLS hypothesis does this role not extend beyond an effect on the frequencies of behavioural forms. As for the source of their behavioural forms, the ape ZLS hypothesis argues that apes’ individual learning (used in a wide sense) contributions to form have been systematically underestimated (Bandini and Tennie 2017, 2019, 2020). According to the ape ZLS approach, individual and social learning work in conjunction^65^ to create an *illusion* of a spread in ape cultures, where the resulting forms remain individually derived and lack cultural evolution (see Fig. 1b). This approach addresses the equifinal (i.e. multifaceted) nature of culture (compare also Barrett 2018) and overall the ape ZLS hypothesis currently provides, we argue, the most appropriate explanation for ape (and some hominin) culture. Indeed, a growing number of studies continue to demonstrate that naïve, ecologically relevant apes, are capable of reinnovating the same behavioural forms as their wild counterparts, without copying of these forms (e.g. Tennie et al. 2008; Lehner et al. 2010; Allritz et al. 2013; Menzel et al. 2013; Reindl et al. 2016; Bandini and Tennie 2017, 2019, 2020; Neadle et al. 2017). If additional work continues to identify reinnovations of forms when subjects are in the appropriate inner and outer circumstances and in the right age, and if apes continue to be restricted to only expressing the forms that they could have individually reinnovated (no copying beyond their ZLS), then this might turn out to be the key difference between human and ape culture (and this view might extend to some hominins, too; Tennie et al. 2016, 2017). Whilst humans can go beyond their ZLS and sustain and create cumulative culture of forms consisting of directly and indirectly copying-dependent forms,^66^ apes may be restricted to ZLS variants of culture that are only influenced in their frequency by non-copying variants of social learning, while the behavioural and artefact forms themselves do not rely on copying (Tennie et al. 2009; Neadle et al. 2017). Although some forms may depend on past individual reinnovations whose frequencies were under social control (grey zone of cumulative culture, compare Tennie et al. 2020) and some frequencies may depend on non-copying social learning (step-wise traditions; Tennie et al. 2009). This view is also in line with the finding that ecologically relevant apes do not seem naturally inclined to copy behavioural forms (Tomasello and Call 1997; Tennie et al. 2012; Clay and Tennie 2017; Bohn et al. 2020). This may be because they lack a native ability or tendency and/or they lack a largely culturally evolved ability to copy in this way (Tennie 2019a; compare Heyes 2018).

Future studies should test more ape forms following the latent solution methodology to examine if any of these forms fail to be reinnovated despite appropriate sample sizes (Bandini and Tennie 2018; Bandini et al. 2020; Neadle et al. 2020). These forms would then warrant special attention^67^ as they might be examples of ape copying-dependent forms—behaviours or artefact forms that go beyond the ape ZLS, or where, instead, there might be non-copying factors that merely hindered reinnovation (such as, perhaps, age of subjects; Neadle et al. 2020; or functional fixedness; Hanus et al. 2011).

Finding consistent evidence for copying-dependent forms in apes would falsify the specific ape ZLS hypothesis, although the ZLS hypothesis would then continue to a) be a possible account for other animal cultures and b) could still serve as an explanation for many or most ape form phenomena (or, in an extreme case (that seems currently unlikely),^68^ for only few forms, similar to the case of humans; Reindl et al. 2016, Neldner et al. 2020). Furthermore, as most previous latent solution type experiments with form-naïve, ecologically relevant apes have been successful, we can conclude that apes apparently do not need to copy their behavioural or artefact forms (though again, the frequency of these forms is often under social mediation control). Therefore, regardless of which variant(s) of social learning are one day determined to be required for cumulative culture, and regardless of whether there are some ape copying-dependent forms in the wild, this major finding remains unaffected: we can logically conclude that the many ape behavioural forms that reappeared spontaneously in such studies do not require copying. In the meantime, given current data, the ape ZLS hypothesis seems to provide an alternative, parsimonious approach to explaining the behavioural and artefact form repertoires observed in apes. Therefore, we argue that the ape ZLS approach should be considered the new null hypothesis for ape behavioural and artefact forms (see also Motes-Rodrigo and Tennie in review). Indeed, the challenge for the ape copying social learning hypothesis is to provide evidence that apes can, but also that they regularly do, transmit forms (causally copy)–especially those behavioural or artefact forms outside of their individual reach (copying-dependent forms).

Kühl et al. (2019) recently reported that human impact reduces the numbers of cultural behaviours shown by chimpanzee populations. Based on this and on general considerations, Kühl et al. (2019) have called for the protection not only of ape genes and ape environments, but also of ape culture. Indeed, one chimpanzee behavioural form, nut-cracking has now been granted UN protected status (Pacheta 2020). To our knowledge this is the very first time this status has been awarded to any non-human primate culture. We are in complete agreement that ape genes and environments need protection, and that their culture should be factored in as well. As we have argued, the typical ape culture is based on socially mediated reinnovations (latent solutions).^69^ While we agree that these ZLS cultures deserve protection, too, we note that any ape culture that has evolved population-specific copying-dependent forms (if such cases exist) would arguably be especially in need of protection—as even the long-term the fate of these forms could then be chained to the fate of their containing populations (compare also Motes-Rodrigo and Tennie, in review).

## Footnotes

1 We define ‘behavioural forms’ as the specific action component(s) of a behaviour, including their organization in a linear and/or hierarchical relationship over time. We define ‘artefact forms’ as the physical component(s) of an artefact, inclduing their organization in a linear and/or hierarchical relationship over time.

2 The most commonly used method for supposedly testing action copying in animals is the “two-target” task (e.g., Custance et al. 2001; Dindo et al. 2008; Kis et al. 2015; Stoinski et al. 2001; Whiten 1998; Whiten et al. 1996, 2005). These tests involve seeding two different techniques into separate groups, to examine whether subjects will use the demonstrated technique more often than the non-seeded technique. The result of these studies across species—and this is not restricted to primates—is that many animals will preferentially show the seeded technique. However, these tasks rarely identify action copying, as the tasks are typically grounded in differences in demonstrated action techniques as well as differences in physical techniques (Heyes and Ray 2000). Furthermore, so far in all tests at least one individual has reinnovated the *non-seeded* technique form as well (Bandini and Tennie 2017, 2019; Tennie et al. 2020), showing that the demonstrated technique forms do not causally require copying social learning, as they can be individually learnt—they are not copying-dependent forms. Thus, these studies only tested the ability for non-copying social learning (or socially mediated (cued) reinnovation of associated latent solutions), but not the presence of action (or other) form copying (Tennie et al. 2006; Bandini and Tennie 2017).

3 Some animal song form specifics likely depends on form copying.

4 The earlier term we used was ‘culture-dependent traits*’.* However, “trait” is arguably a too general ‘umbrella’ term for targets of social learning, which may, for example, instead simply include locations or various types of stimuli (i.e. as targets). Once exposed to these locations or stimuli, the observer may then become more interested/likely to explore these—simply as a result of non-copying social learning mechanisms (in the example here: local/stimulus enhancement). Thus, and whilst it is possible that social learning may even be required to lead to this outcome, e.g., in a socially mediated ‘re-discovery’ of a well-hidden location such as, say, a perfect water-hole hidden in a cave behind a bush (“step-wise traditions” in Tennie et al. 2009), we would like to meaningfully distinguish such culture-dependent traits from culture-dependent—and, more precisely, copying-dependent—*form* (e.g., of artefacts and/or behaviour whose form (designs) depend on copying social learning—such as, e.g. tango dance steps or ways to build a rocket). In a way, the form that we highlight might be equated with know-how—expressions of know-how—whereas other social learning mechanisms target other types of knowledge (e.g. know-where (e.g. local enhancement), know-what (e.g. stimulus enhancement) etc. (see also Tennie et al. 2017; Bandini et al. 2020)).

5 And we know this because there were, for example, no airplanes a mere 120 years ago.

6 The ZLS hypothesis may be seen as a (positive) answer to the question whether and/or how different social learning mechanisms may matter in cultural evolution (cf. open discussion on the last page of Lewens’ (2018) cultural evolution entry in the Stanford Encyclopedia of Philosophy).

7 i.e. through non-form-copying variants of social learning such as local enhancement and exposure etc.

8 Though note that there can also be cases where latent solutions are readily and universally expressed by all individuals (in a given environment), creating a ceiling effect. In such cases (e.g. general preference for being active during daylight, or even breathing behaviour), social learning may not create an additional or differential or generally important effect. These cases would be *closer* to what classically may have been described as instincts (though we prefer not to use, let alone restrict us to, this laden and out-dated term; as we have also laid out clearly in Bandini and Tennie 2018 contra claims by Haidle and Schlaudt 2020).

9 Of course, universally expressed traits can be influenced by social learning mechanisms, too (the field has known this since Laland and Janik 2006 seminal paper).

10 See Reindl et al. (2020) for a baseline version of this study, additionally differentiating between two possible formulations of the cumulative culture concept.

11 Under special circumstances, cumulative cultural evolution can also be due to cumulating copying error alone (Eerkens and Lipo 2005)—but of course this requires form copying skills in the first place.

12 Though there can also be serendipity and recombination; not represented in our particular game here (Muthukrishna and Henrich 2016).

13 As used, e.g. by Stout et al. (2019), Morin (2016) and Charbonneau (2018).

14 One other difference being that wild ape populations are cluster-connected and not linear as in our example. However, the underlying logic remains the same regarding a lack of direct form evolution.

15 The reinnovations process itself in real life is far from simple, and will usually depend on a complex interaction of several factors, such as genetic setup, cognitive skills and environmental conditions etc. Therefore, there are in reality “several whisperers” that cannot be meaningfully separated. Yet, regardless of this complexity underlying reinnovation (always highlighted in the ZLS approach), the empirical evidence in apes is that our focus here—the output form of the reinnovation—remains comparable across individual apes (see “similarity gambit” below).

16 Of course, this metaphor should not be taken too far: the fact that a single person whispering in each ear carries a copying-dependent message form is obviously a deviation from the real-life case of latent solutions. Closer to real life would be a game in which each player would have their own whisperer(s), and after listening, participants may tap the shoulder of the whisperer of the next person to make these whisper etc. (so there would be two rows of people (players and whisperers) in a full version of a latent solution game). Note also that in our example we only implemented one signal type (a tap)—but several differentiating signal cues could be used where each socially mediates (cues) a different reinnovation form (e.g. a tap on the nose could trigger the whispering of a *different* sentence form in our game).

17 Some ape cultures may not exist without this socially-mediated reinnovation. Such cases are in some way frequency-dependent reinnovations—or step-wise traditions (Tennie et al. 2009).

18 And so, stasis is not a good indicator of form copying. Indeed, stasis is to be expected in the *absence* of form copying. While various factors (see below) can sometimes hide copying effects, stasis can therefore often be used as a sign for an absence of form copying. Note that Gruber (2020) misrepresents these arguments, by alleging instead that Tennie et al. (2017) claim that stasis is or must be due to form copying. This is not the case—in fact it goes diametrically against the entire stance that was put forward by Tennie et al. (2017). We imagine that Gruber intended to cite external commentators (Gowlett and de la Torre) to the 2017 target article by Tennie et al. This would make sense, as some of these commentators (but not the authors of the target article) indeed suggested such a (questionable, and likely wrong) causal link between stasis and the presence of copying.

19 For example, the fact that the forms of early sharp stone tools eventually changed often (albeit very slowly; Tennie et al. 2016, 2017) shows that there is no inherent, always perfect form hidden inside stones per se (though certain forms may have presented optima to specific goals and associated constraints at their times and for their makers).

20 A misleading term when it comes to the difference between form-copying and non-form copying social learning mechanisms, see Tennie et al. (2020). It will therefore be avoided here.

21 Provided appropriate subjects have been tested (i.e., form-naïve individuals). Examples include Huffman and Hirata (2004), Köhler (1925), and Köhler and Winter (1925).

22 The ZAD describes what human *A* can do at time *X*. Once the ZPD (plus maturation) has further impacted on human *A*, then his/her ZAD will have moved—it usually will have become further enlarged (at time *X + *1). ZPD effects can be very large in the human case, but are much lower, if present at all (when effects of form-copying are considered), in the case of apes (see similarity gambit below).

23 The innovative reach of a given species can be studied by carefully designed reinnovation experiments in form-naïve, ecologically relevant subjects and can, in this way, be quantified over time. There are even calculations that help determine at which point it can be assumed that a given innovation may be outside the species’ reach (see Bandini and Tennie 2018; Tennie et al. 2020).

24 Form copying abilities enable increases in the number of available forms to individuals, which can lead to a veritable explosion of form possibilities—a major engine of the ZPD-ZAD axis (see also section “ZLS as related to cultural epidemiology” below).

25 See also Tennie et al. (2020) for a general critique of the concept of high-fidelity versus low-fidelity social learning in the light of the difference between form-copying and non-form-copying.

26 The relative stasis in the early stone tool archaeological record suggests that the similarity gambit extends to the hominins producing these tool forms—which means that the ZLS application is justified for these hominins, too—and that it should be the null hypothesis for their stone tool production (Tennie et al. 2016, 2017).

27 Including the possibility that individual A is just less ‘lucky’ than the innovator, who might simply found themselves, stochastically, in the right conditions (e.g. next to a bees nest) to innovate the behavioural form (Bandini et al. 2020)—but also including the possibility of a somewhat relatively lower cognitive power that needs to be bridged (but can be bridged) by socially mediated reinnovation.

28 Although note that Bandini and Tennie (2017) originally described *two* standards, based on the relative human-perceived complexity of the target behavioural form—this was introduced purely to increase validity (and is not an absolute necessity). The more conservative standard is applied for relatively simple behaviours and requires two independent reinnovations of the behaviour for it to be considered within the ZLS (this is the double-case ZLS standard). For more complex behaviours, such as chimpanzee nut-cracking (which involves a set of predetermined steps and more than one tool) this approach would only require one single reinnovation to make the claim that the behavioural form is in the ZLS of the species, as these behaviours should be less likely to have been reinnovated by chance (this is the single-case ZLS standard).

29 There will always be some who question whether subjects really have not seen the target form being performed before testing. Yet, given that ecologically relevant apes are unable to copy novel behavioural forms (see above), technically one could even do without this requirement (as it should not matter). Thus, this requirement merely serves as an additional, but likely redundant, control.

30 These tests are usually performed in a captive setting, though they can also be performed in the wild.

31 Similarly, young capuchins take years to master nut-cracking but this, too, may be due to other factors such as the strength, skill and manual dexterity the behaviour demands—which all may simply require time to individually mature (de Resende et al. 2008).

32 Unfortunately, we have very little data on these original ape innovators (for an exception see Hobaiter et al. 2014; who provide evidence of *several* independent innovators when in a naturally occurring (rare) situation of condensed material proximities). The rarity of these situations may also help explain why some latent solutions are not demonstrated in all sites in which the raw materials exist.

33 In essence, in the wild the objects are made salient amongst a background of other objects, whereas in these tests we may sometimes enhance these objects by removing background.

34 For apes, this would include the likelihood that they had observed the keepers or visitors showing the target behaviour. This likelihood is established by asking current and past keepers of the animals and by checking whether keepers or visitors could conceivably have shown the behaviour in front of the apes. However, again, and unlike in the case of modern humans, this check actually matters less for the apes—as in all likelihood apes do not spontaneously copy novel actions they observe (see main text).

35 In the human case, such systematic genetic differences do not exist and can thus be ignored.

36 Note that we are not here referring to the *use* of these tools—there are, for example, single-button emergency pagers, whose streamlined use might not even be completely beyond other primates’ abilities.

37 An additional question—but not the focus of the ZLS approach—could then be why the various forms appear. But what would be of interest for the ZLS approach would be if there are gene- and environment independent cues that lead to socially mediated reinnovation specifically for the various resulting forms (i.e. the question of the specific “tapping” cues in Fig. 1b).

38 We prefer social learning biases, as the term transmission already implies copying of form (but which is rare in animals).

39 This evolution happens both directly, and indirectly, via effects that can also derive from mere ZLS cultural dynamics (for details, see van Schaik and Pradhan 2003 and Tennie and Over 2012; and for the human case, involving even the cultural evolution of whole cognitive mechanisms, see Heyes 2018). The general framework here is a kind of Baldwin effect/genetic assimilation/cultural drive within a triple inheritance framework (where subjects inherit genes, environment and culture but where these levels all co-evolve). Although note that currently we do not know the timescales in which this evolution occurs. Timescale of change is a largely an open question (including for the ZLS hypothesis) that we intend to investigate in future (though see Boyd et al. 2013 for some related discussion). Note also that changes need not be unidirectional, either.

40 Indeed, both form-naïve chimpanzees and orangutans can solve human-inspired tasks (Herrmann et al. 2007) while form-naïve humans can solve orangutan- and chimpanzee-inspired tasks (Reindl et al. 2016; Neldner et al. 2020).

41 For example, we may see these when we test across species and find similar behaviours appear—without any evidence for precursors in that species’ past and without a correlate in the wild.

42 Perhaps due in part to cultural drive effects; though note that cultural drive can happen also without form copying mechanisms being present (in which case we might call it *minimal cultural drive*).

43 As White and Ashton (2003) note, these reinnovations might have already required a pre-existing background (Acheulean techniques) and so, these cases might fall into the grey zone of cumulative culture (see Tennie et al. 2020) or into an effect of the collective brain hypothesis—and/or into phylogenetic cultural intelligence (see main text).

44 Which also implies that these mechanisms and biases culturally evolved—in the hominin lineage only (the question is: when this happened; see Tennie et al. 2016, 2017 for discussion that suggests that this happened late).

45 Also often referred to as cultural attraction theory (CAT).

46 He continued that “the next, critical step is to identify the various factors, cognitive or otherwise, that determine why it is these items, and not others, that are easily re-producible.” While these are important questions, they are not a necessary extension that must follow for the ZLS approach.

47 That is, the modern “extended” variant of CE (compare Acerbi and Mesoudi 2015).

48 Though, as Acerbi and Mesoudi (2015) note, this viewpoint, or the “narrow sense of CE”, could then perhaps be subsumable under the (academically older) concept of “guided variation” of gene-culture coevolutionary theory (last summarised in Richerson and Boyd 2005). Note also in this context that—in contrast to CE—Richerson and Boyd do *not* argue that guided variation is of outstanding importance in modern humans.

49 Seen in this way, triple inheritance theory and CE are somewhat aligned together, at least with regards to an overall framework of factors. We too promote and use triple inheritance as the most appropriate general framework (see above).

50 Buskell (2019) points out that CE typically focuses on ultimately nativist factors instead (one can see this, e.g. in Sperber 2000). This limits CE’s applicability (see also below).

51 Given that several, interacting and dynamic factors are utilised by CE, the critique of Boyd et al. (2013)—portraying CE as a purely nativist (~ fixed) view that could only ever explain one unchanging set of forms within a given (and unchanging) environment—does therefore not fully apply, or does at least not apply to current, extended versions of CE (compare Acerbi and Mesoudi 2015).

52 Both promote the idea of input-form independent output-forms, or short, copying-*independent*-forms.

53 A more specific variant of environment, artefact form, can best be seen as essentially cultural, too—and so will not be considered separately.

54 All that would seem to be required for this is another mental arrow going back also to the old constructor and who would likewise attend to the form in question.

55 This can also be a mirrored resemblance (as in the genetic code (A to T; G to C etc)—the key point being that the output form must causally and statistically correspond to the input form.

56 The ZLS does not focus on mental forms, simply because they cannot be well quantified in the case of apes (and even in the human case this is difficult).

57 Thanks to A. Buskell for the example.

58 In animals, a comparable finding exists for the re-occurrence, but only over several generations, of wild-type song patterns in a song-bird cultural transmission experiment (Fehér et al. 2009).

59 This could be another deviation from the ZLS—in the ZLS there is and can be form reinnovation *on the spot*, entirely without the specific input present.

60 Imagine one copies a book with a special “photocopier” but that merely detects page numbers to then print the corresponding pages out from its memory. The illusion of complete form copying would be strong to the person using this machine (note that this situation again corresponds to our Fig. 1b). This would seem to be the view of Sperber’s concept of “triggering” (2000), and corresponds to our use of socially mediated reinnovation as cues for specific form reinnovation.

61 Indeed, it did not stop there. Both researchers influenced others, but also each other—and even themselves again. For example, Darwin was later influenced by himself whenever he produced new, updated editions based on earlier instances of his publications. We recommend Nettle (2018) as an insightful and humoristic exploration of these complexities.

62 And/or that both views get somewhat merged.

63 Note that this view—effects of direct and indirect form copying also on innovative/cognitive skills—resembles somewhat recent views of Daniel Dennett—who uses a “meme’s eye approach” to explain human cognition (Dennett 2017). In it, Dennett also explores additional complications we could not get into detail here (e.g. nested hierarchies of forms). In a longer piece we would also have linked more thoroughly to accounts that view cultural transmission being responsible even for much of the underlying copying and cognitive human machinery itself—as so-called “cognitive gadgets” (Heyes 2018).

64 See Tennie 2019a, for a first discussion on the lack of lasting effects of human enculturation on apes’ cognition and culture across ape generations (i.e. for an empirical lack of evidence for the possibility of a Planet of the Apes situation arising, even when kickstarted by humans).

65 Along with other factors such as pre-existing latent solutions in a population, the environment and genetic background, etc. see Bandini and Tennie (2018) and Tennie et al. (2020).

66 Perhaps an ability itself fuelled by cumulative culture (a cognitive gadget, sensu Heyes 2018).

67 Including perhaps at least one retest in a different population—as there can also be (non-copying) factors that *hinder* specific reinnovations in affected populations (see e.g. Tennie and Hopper (2011) online comment on Gruber 2011).

68 If for some reason *most* ape forms turn out to be copying dependent (a situation that seems very unlikely at the moment, given all the positive evidence for the ape ZLS; see main text).

69 Apes clearly deserve to be able to express a wide range of behavioral forms—and they deserve to do this now. Thus, whilst it is true that latent solution-based cultures can eventually reappear again (other than in certain cases in which aspects of the environment are needed but where these fail to recover), this may take substantial time in the wild.
